# Comparative Analysis of Membrane Lipids in Psychrophilic and Mesophilic Freshwater Dinoflagellates

**DOI:** 10.3389/fpls.2016.00524

**Published:** 2016-04-20

**Authors:** Andrea Anesi, Ulrike Obertegger, Gert Hansen, Assaf Sukenik, Giovanna Flaim, Graziano Guella

**Affiliations:** ^1^Bioorganic Chemistry Laboratory, Department of Physics, University of TrentoTrento, Italy; ^2^Fondazione Edmund Mach, Research and Innovation CentreTrento, Italy; ^3^Department of Biology, University of CopenhagenCopenhagen, Denmark; ^4^Kinneret Limnological Laboratory, Israel Oceanographic and Limnological ResearchMigdal, Israel; ^5^Biophysical Institute, Consiglio Nazionale delle RicerchePovo, Italy

**Keywords:** membrane lipids, freshwater dinoflagellates, cold adaptation, RPLC-ESI-IT-MS, multivariate data analysis

## Abstract

Here we report the lipid profiles of ten dinoflagellate species originating from different freshwater habitats and grown at 4, 13, or 20°C akin to their natural occurrence. Lipids were determined by High Performance Liquid Chromatography-ElectroSpray Ionization-Mass Spectrometry in positive and negative ion modes. Besides the well-studied monogalactosyldiacylglycerol (MGDG) and digalactosyldiacylglycerol (DGDG) lipids, our study revealed the presence of intact molecular lipid species of trigalactosyldiacylglycerols, betaine diacylglyceryl-carboxyhydroxymethylcholine, sulfolipid sulfoquinovosyldiacylglycerols (SQDG) and phospholipids, in particular phosphatidylcholine, phosphatidylethanolamine and phosphatidylglycerol. In multivariate ordination, the freshwater dinoflagellates studied could be distinguished into two groups based on their lipid profiles. *Peridinium aciculiferum*, *Borghiella dodgei*, *B. tenuissima* and *Tovellia coronata* belonged to group 1 while *Ceratium cornutum*, *Gymnodinium palustre*, *Jadwigia applanata, P. cinctum*, *P. willei*, and *P. gatunense* belonged to group 2. Indicator species analysis evidenced that group 1 was characterized by 36:9 MGDG and 36:9 DGDG and group 2 by 38:9 and 38:10 MGDG, 38:9 and 38:10 DGDG and 34:1 SQDG. We suggest that the grouping of dinoflagellates indicated their range of temperature tolerance. Furthermore, non-thylakoid lipids were linked to dinoflagellate phylogeny based on the large ribosomal sub-unit (28S LSU) rather than their temperature tolerance. Thus certain lipids better reflected habitat adaptation while other lipids better reflected genetic diversity.

## Introduction

In marine and freshwater ecosystems, photosynthetic dinoflagellates are important primary producers, both as free-living cells and as symbionts (Leblond et al., 2010b). Ectothermic organisms, including dinoflagellates, are especially susceptible to climate change and their biogeographical distribution depends on physiological, biochemical and molecular mechanisms governing species temperature tolerance (Somero et al., 1996; Thornhill et al., 2008; Somero, 2012). Mechanisms of temperature tolerance in ectotherms have been substantially linked to proteins via enzyme activity and membrane integrity (Somero et al., 1996). Lipids, however, have received less attention, in part because unlike proteins they have no recognizable catalytic properties, and thus it is impossible to determine directly the function of lipids *in vitro* (Murata and Siegenthaler, 2004). Lipids are an essential part of membranes, and modifications of membrane lipid composition and architecture in response to environmental conditions are known as *homeoviscous* adaptation (Hazel, 1995). Common strategies to maintain adequate membrane fluidity involve the incorporation of polyunsaturated fatty acids, the position and conformation of double bonds, the length of fatty acyl chains and the presence of sterols (White et al., 2000; Chintalapati et al., 2004; D’Amico et al., 2006; Guschina and Harwood, 2006a,b). The incorporation of methyl branched fatty acids, carotenoids and the modification of polar head group composition are less widespread (D’Amico et al., 2006).

Photosynthetic organisms need to compensate changes in thylakoid membrane fluidity as determined by environmental conditions (e.g., temperature, nutrients) to prevent the inhibition of photosynthesis (Murata, 1989). The active restructuring of membranes with respect to temperature has been studied in different photosynthetic organisms such as cyanobacteria (Wada et al., 1990; Gombos et al., 1992), green algae (Terrados and Lopez-Jimenez, 1996; Valledor et al., 2013), diatoms (Chen et al., 2008, 2013; Dodson et al., 2014) and higher plants (Routaboul et al., 2000; Welti et al., 2002, 2007). In dinoflagellates, most of what we know about the active restructuring of membranes with respect to temperature refers to marine species (e.g., Mansour et al., 1999; Leblond et al., 2006; Gray et al., 2009a,b; Dahmen et al., 2013), and few studies have investigated temperature related changes in freshwater dinoflagellates (Flaim et al., 2012, 2014). Furthermore, studies usually focus on either cold-adapted (Flaim et al., 2014) or warm-adapted species (Gray et al., 2009a,b; Leblond et al., 2010a).

Membrane lipids are a mixture of neutral and anionic lipids. Neutral lipids comprise monogalactosyldiacylglycerols (MGDGs), digalactosyldiacylglycerols (DGDGs) and, in some algae such as diatoms and dinoflagellates, trigalactosyldiacylglycerols (TGDGs). MGDGs and DGDGs are the most abundant lipids in nature (Siegenthaler, 2004), accounting for about 50 and 30% of total lipids in chloroplast membranes respectively, and they exert structural and functional roles (Murata and Siegenthaler, 2004; Siegenthaler, 2004). TGDGs have been suggested to act as viscosity buffers (Gray et al., 2009a), are fatty acid carriers (Xu et al., 2010; Moellering and Benning, 2011) and seem to support thylakoid function irrespective of temperature (Flaim et al., 2014). Anionic lipids comprise the phospholipid phosphatidylglycerol (PG) and the glycolipid sulfoquinovosyldiacylglycerol (SQDG). PGs are also important for the structure and activity of photosystem II that is embedded in the lipid environment of the thylakoid membranes of plants and algae (Barber, 2012), and PG’s function can be replaced by SQDG under phosphorus limitation (Yu and Benning, 2003). PG and SQDG account for about 5–12% of total lipids.

Apart from thylakoid membranes, polar lipids also occur in plasma membranes and extra-chloroplast membranes; presently, little information is available about these lipid fractions regarding temperature adaptation; in dinoflagellates and other algae these polar lipids comprehend the phospholipids phosphatidylcholine (PC) and phosphatidylethanolamine (PE) and betaine lipids (Siegenthaler, 2004). PC is usually the most abundant phospholipid in eukaryotic membranes, followed by PE. Betaine ether-linked lipids are present in a limited number of species, mainly in algae such as dinoflagellates, ferns, lower plants, and fungi (Sato, 1992; Dembitsky, 1996; Rozentsvet, 2004; Leblond et al., 2015). Three types of betaines are known: diacylglyceryl-trimethyl-homoserine (DGTS), diacylglyceryl-hydroxymethyl-trimethyl-β-alanine (DGTA) and diacylglyceryl-carboxyhydroxymethylcholine (DGCC) (Sato, 1992; Siegenthaler, 2004). Betaines are considered primitive lipids, as they can play the same role in the cells of lower plants as PC. Curiously, an inverse relationship between PC and DGCC has been observed: betaines, which are structurally similar to PC, tend to replace PC in phosphorous-limiting conditions (Klug and Benning, 2001; Popendorf et al., 2013).

Cold-adaptation in dinoflagellates has been the subject of recent studies, with a particular focus on galactolipids MGDG and DGDG. By using gas chromatography-mass spectrometry (GC-MS) methodology, Leblond et al. (2006) found octadecatetraenoic 18:4 (ω3) and octadecapentaenoic 18:5 (ω3) acids in high percentages in the cold-adapted dinoflagellates *Peridinium aciculiferum* and *Scrippsiella hangoei*. Investigating 35 peridinin-containing dinoflagellates grown at 21°C, Gray et al. (2009b) found that they clustered into two groups based on the regiochemical distribution of their fatty acyl chains in MGDG and DGDG. Cluster 1 was characterized by the ω3 fatty acyl chain ratio C_18_/C_18_ (18:4 and 18:5 acyl chains at the *sn*-1 and *sn-*2 positions of the glycerol backbone) whilst cluster 2 by the ω3 fatty acyl chain ratio C_20_/C_18_ (20:5 at the *sn*-1 and 18:4 or 18:5 at *sn*-2). Gray et al. (2009b) did not find a clear relationship between galactolipid profiles and phylogeny and suggest that this grouping could be linked to habitat characteristics. A further study (Gray et al., 2009a) on four cold-adapted dinoflagellates (*Gymnodium* sp., *Scrippsiella hangoei*, *Woloszynskia halophila*, and *P. aciculiferum*) found that these psychrophiles (microorganisms with optimum growth below 10°C and maximum temperature tolerance below 20°C; Morgan-Kiss et al., 2006) all belong to the C_18_/C_18_ cluster *sensu* Gray et al. (2009b). An extensive lipid profile encompassing galactolipids, phospholipids, betaines and triacylglycerols (TAGs) for the cold-adapted dinoflagellate *P. aciculiferum* showed that the basic lipid composition was conserved in the vegetative stages (2.5–5.5°C temperature range), while significant changes in the lipid profile were noted at 7°C, a temperature that induced encystment (Flaim et al., 2014). Furthermore, increasing the growth temperature determined an overall decrease in the UI in all lipid classes for this species (Flaim et al., 2014). The psychrophilic *Borghiella dodgei* also showed similar behavior (Flaim et al., 2012); therefore Flaim et al. (2014) suggest that strict psychrophiles possess a limited temperature tolerance before relevant changes in lipid composition occur.

Here we present lipid profiles of ten freshwater dinoflagellate species grown at specific temperatures akin to their natural occurrence. We focused on three psychrophiles (*P. aciculiferum*, *B. dodgei*, *B. tenuissima*), six temperate dinoflagellates (*C*. *cornutum*, *G*. *palustre*, *J*. *applanata*, *P*. *cinctum*, *P*. *willei*, *T*. *coronata)* and one warm-water dinoflagellate (*P. gatunense*). The psychrophiles all occur in very cold waters in winter or early spring (Moestrup et al., 2008; Flaim et al., 2012, 2014). *Peridinium gatunense* occurs in sub-tropical lakes in spring and in temperate lakes in summer (Hansen and Flaim, 2007; Zohary et al., 2014) while the others generally have broader temporal and spatial occurrence (Hansen and Flaim, 2007). We investigated the association of these freshwater dinoflagellates with the C_18_/C_18_ and C_20_/C_18_ clusters *sensu* Gray et al. (2009b) and linked their lipid profiles to their growth temperature and genetic diversity.

## Experimental

### Chemicals

HPLC grade methanol and chloroform were purchased from VWR (VWR International, Milan, Italy); LC-MS grade methanol was purchased from Merck (Merck KgaA, Darmstadt, Germany). Deionized water, filtered at 0.2 μm, was obtained from Elix Water Purification System (Merck Millipore, Billerica, MA, USA). MGDG and DGDG standards were purchased from Matreya LLC (State College, USA).

### Dinoflagellate Selection and Culture Conditions

Ten freshwater dinoflagellates were grown at temperatures that reflected their temperature niche and permitted active growth (**Table 1**).

**Table 1:** Freshwater dinoflagellate species used in lipid profiling; origin of cultures, growth media and growth temperature are given; Scandinavian Culture Collection of Algae and Protozoa (SCCAP; http://www.sccap.dk/); Kinneret Limnological Laboratory culture collection (KLL; Zohary et al., 2014).

species	origin	growth medium	growth temperature
*Borghiella tenuissima*	SCCAP K-0666	DY-V	4°C
*Borghiella dodgei*	SCCAP K-0959	DY-V	4°C
*Ceratium cornutum*	SCCAP K- 1412	DY-V	13°C
*Gymnodinium palustre*	SCCAP K-0995	DY-V	13°C
*Jadwigia applanata*	SCCAP K-0677	DY-V	13°C
*Peridinium aciculiferum*	SCCAP K-0998	MWC+Se	4°C
*Peridinium cinctum*	SCCAP K-1382	MWC+Se	13°C
*Peridinium gatunense*	KLL	L16-V	20°C
*Peridinium willei*	SCCAP K-0962	DY-V	13°C
*Tovellia coronata*	SCCAP K-1118	DY-V	13°C


*The growth media, MWC + Se and L16-V are modifications according to Guillard and Lorenzen (1972) and Lindström (1991), respectively. DY is according to Andersen et al. (1997); all media recipes can be found at http://www.sccap.dk/media/.*

Light intensity was approximately 20–40 μmol m^-2^ s^-1^ [measured at the culture surface using a Quantum Photo Radiometer (Delta Ohm srl, Caselle di Selvazzano, PD, Italy)] with a 14:10 light:dark cycle for all dinoflagellates except *P. gatunense* (12:12). Cells were grown in batch cultures and harvested by centrifugation at 3000 × *g* for 10 min at their growth temperature at the end of the exponential phase.

### Total Lipid Extraction

Cells were washed three times with distilled water and the final pellet was extracted with a 1:1 chloroform: methanol (v:v) mixture using a glass/glass potter on ice, checked microscopically for complete cell disintegration, and collected into 15 mL glass tubes; re-suspended in 10 ml of chloroform/methanol 2:1 (v/v), sonicated for 15 min in an ultrasonic bath (Sonorex Super, Bandelin electronics, Berlin, Germany), and centrifuged at 3000 × *g* for 10 min at room temperature to separate the organic phase (bottom layer). All the organic phases were collected, filtered by using Whatman GF/X filters (0.45 μm) under vacuum and reduced to dryness on a rotary evaporation (Büchi Labortechnik AG, Flawil, Swiss) to obtain crude lipid extracts. Extracts were re-suspended in 300 μL of methanol.

### Reverse Phase Liquid Chromatography-Electrospray Ionization-Ion Trap-Mass Spectrometry Analyses (RPLC-ESI-IT-MS)

Liquid chromatography-electrospray ionization-mass spectrometry allows separation of a wide variety of intact lipid molecular species and gives detailed structural information about lipid head groups and the FAC regiochemical distribution (Welti et al., 2002, 2007; Guella et al., 2003; Anesi and Guella, 2015). To analyze crude lipid extracts we used a Hewlett-Packard Model 1100 Series liquid chromatograph (Hewlett-Packard Development Company, L.P., Palo Alto, CA, USA) coupled both to a Bruker Esquire-LC quadrupole IT-MS equipped with an ESI source (Bruker Optik GmbH, Ettlingen, Germany) and to a photo diode-array detector (DAD) (Agilent Technologies, Milan, Italy, Agilent 1100). Chromatographic separation of lipids was carried out on a Zorbax Eclipse XDB-C8 column (150 × 4.6 mm i.d., pore size 200 Å, particle size 3.5 μm) (Hewlett Packard, Palo Alto, CA, USA) with a linear gradient of solvent A (methanol: water 7:3, containing 12 mM ammonium acetate) and solvent B (methanol containing 12 mM ammonium acetate) from 70%A/30%B to 100%B in 40 min, at a constant flow rate of 0.8 ml/min. Final conditions were kept for at least 30 min to ensure the complete elution of non-polar lipids. Aliquots of 10 μL of crude extract in methanol-*d*_4_ were injected.

Each crude extract was separately analyzed in positive and negative ionization modes in the range 50–1200 *m/z* with a scan range of 13000 unit s^-1^. For the analysis, high purity nitrogen was used at a pressure of 35 psi, at a temperature of 300°C and a flow rate of 7 L min^-1^. The high voltage capillary was set at 4000 V for positive ionization mode and -4000 V for negative mode. The regiochemical distribution of acyl chains was conducted through MS/MS experiment in positive and negative ion as described by Guella et al. (2003) for galactolipids and by Flaim et al. (2014) for betaines and phospholipids. We applied a recently published method (Anesi and Guella, 2015) based on HILIC chromatography coupled to precursor ion/neutral loss scanning in positive ion mode to improve the assignment of lipid molecular species.

### Relative Quantification of Lipids and Data Analysis

Raw data were analyzed by DataAnalysis 3.0 software (Bruker Daltonik, Ettlingen, Germany). Each lipid molecular species was quantified with respect to the total area of all lipid species belonging to the same class (e.g., relative quantification of PC was performed with respect to total area of PC). MGDG, DGDG, TGDG, DGCC and PC, were quantified on a dataset recorded in positive ion mode, while PE, PG and SQDG on that obtained in negative ion mode. In particular, ESI(+) MS response factors for MGDG and DGDG were established by the use of the commercially available internal standard (MGDG 18:3/18:3 and DGDG 18:3/18:3) (Flaim et al., 2014).

For statistical analyses, the UI and the ACL were calculated for each lipid class, using the formulas

$$U\,I_{c\,l\,a\,s\,s\,\;y}=\Sigma \,\left(r\,e\,l\,a\,t\,i\,v\,e\,\;a\,r\,e\,a\,\;l\,i\,p\,i\,d_{x}*d\,o\,u\,b\,l\,e\,\;n\,u\,m\,b\,e\,r\,\;o\,f\,\;l\,i\,p\,i\,d_{x}\right)\,\;a\,n\,d\,\;A\,C\,L_{c\,l\,a\,s\,s\,\;y}=\Sigma \,\left(r\,e\,l\,a\,t\,i\,v\,e\,\;a\,r\,e\,a\,\;l\,i\,p\,i\,d_{x}*a\,c\,y\,l\,\;c\,h\,a\,i\,n\,\;l\,e\,n\,g\,t\,h\,\;o\,f\,\;l\,i\,p\,i\,d_{x}\right)$$

where lipid*_x_* represents each single molecular species belonging to the *y* lipid class, respectively.

The ratio between MGDG and DGDG was calculated as [Σ area MGDG/Σ area DGDG] ^∗^ F; where F is the normalization ratio obtained by using internal standard (Flaim et al., 2014).

Data were log(x + 1) transformed and scaled using the Pareto method. At first, we applied a PCA (an unsupervised method) to define homogeneous clusters of taxa based on % area of single molecular species, UI and ACL.

Then, we used the identified clusters as dependent variables first in PLS-DA followed by OPLS-DA, both supervised methods. Significance of PLS-DA was determined with permutation tests (200 permutations). PCA and DA were performed with Simca-P 13.0 software (Umetrics AB, Umea, Sweden). We furthermore analyzed the correlation loading plots of OPLS-DA analysis to determine which metabolites contributed to the separation of clusters by setting a correlation coefficient p(corr) threshold of 0.75; a two-tailed Welch test for single candidate markers was carried out to investigate their status as markers; unequal variance between groups was considered.

Similarly to Leblond et al. (2010b), we investigated the relationship between the dinoflagellates’ phylogeny and their lipid profile by calculating the correlation between the dissimilarity matrix based on uncorrected genetic distance and Euclidean distance based on the fatty acyl chains. The uncorrected genetic distance was calculated based on a 606 base pairs long part of the 28S LSU gene downloaded from GenBank (accession number: *B. dodgei* EU126801, *C. hirundinella* JQ639749 (in substitution of *C. cornutum* that was not available in GenBank), *J. applanata* AY950448, *P. aciculiferum* EF417312, *P. cinctum* EF205011, *P. gatunense* EF058267, *P. willei* AF260384, AF260384, *T. coronata* AY950445, *B. tenuissima* AY571374, *G. palustre* AF260382) where all species overlapped. The correlation between matrices was done by Pearson correlation and its significance was assessed by a Mantel test. We, furthermore, investigated the relationship between dinoflagellate species based on their lipid profile by combining a cluster analysis with Non-Metric Multidimensional Scaling (NMDS). While a cluster analysis searches for discontinuities in the data, ordination techniques such as NMSD extract the main patterns, and combining them can be an advantageous approach to investigate differences between groups (Borcard et al., 2011). In NMDS, we used Euclidean distance and in hierarchical clustering, we used Ward’s clustering method based on correlations. In NMDS, the goodness of fit was investigated by the Shepard plot that shows the relationship between the inter-object distances in NMDS and Bray–Curtis dissimilarity. The residuals of this relationship were used to calculate Kruskal’s stress (S); *S*-values <0.2 are considered statistically meaningful (Quinn and Keough, 2002). In hierarchical clustering, significant clusters were identified by bootstrap resampling (Suzuki and Shimodaira, 2015). Hierarchical clustering, calculation of uncorrected genetic distances, Mantel tests and NMDS were performed with R (R Core Team, 2014), package vegan, ape and pvclust.

## Results

Overall, we found 32 galactolipid species (9 MGDGs, 13 DGDGs, 10 TGDGs), nine SQDGs, 29 DGCC and 25 phospholipids (19 PCs, 3 PGs, 3 PEs), whose distribution is taxa-specific (see **Supplementary Table S1** for detailed information).

**FIGURE 1:**
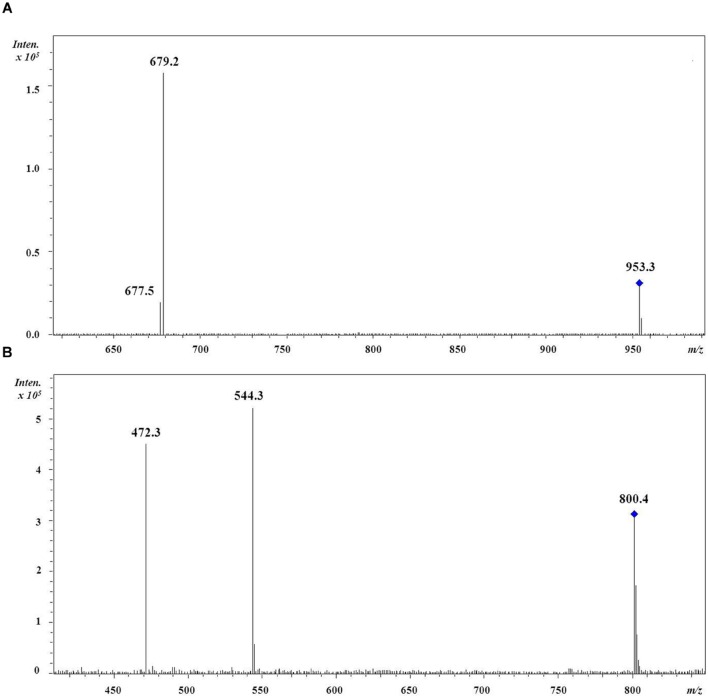
**Positive-ion MS/MS spectra of **(A)** DGDG (18:5/18:4).** The fragment at *m/z* 679 represents the loss of the *sn*-1 linked acyl chain 18:5 whilst the fragment at *m/z* 677 represents the loss of the *sn*-2 linked acyl chain 18:4; **(B)** DGCC (16:0/22:6). The fragment at *m/z* 544 represents the loss of the acyl chain 16:0 while the fragment at *m/z* 472 represents the loss of the acyl chain 22:6 but no information about their regiochemical distribution is implied.

**FIGURE 2:**
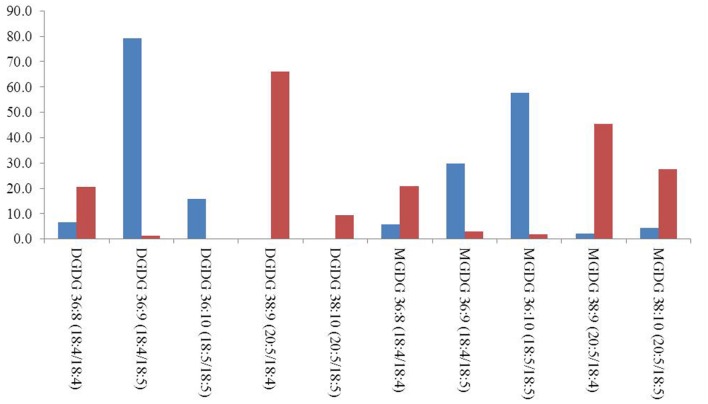
**Relative distribution of most abundant MGDG and DGDG molecular species in *B. tenuissima* (blue) and *C. cornutum* (red) dinoflagellates.** Only species above 2% were considered.

The regiochemistry of acyl chains for most abundant MGDG and DGDG species was established by the method proposed by Guella et al. (2003). As shown in **Figure 1A** for the 36:9 DGDG [M+Na]+ molecular species, in positive ion-mode the fragments at *m/z* 679 and 677 represent, respectively, the loss of the acyl chain 18:5 and 18:4 from glycerol backbone. Since, according to Guella et al. (2003), the loss of the carboxylic acid linked to the *sn*-1 glycerol position always produces a more intense peak (here at *m/z* 679) than that derived from the loss of the *sn*-2 linked acyl chain, the regiochemical distribution of this 36:9 DGDG species can be unambiguously assigned as 18:5/18:4. In all dinoflagellates the 36:8 (18:4/18:4), 36:9 (18:5/18:4), 36:10 (18:5/18:5), 38:9 (18:4/20:5) and 38:10 (18:5/20:5) were the most abundant species of MGDGs and DGDGs. Taken as an example, the distribution of MGDG and DGDG in the cold adapted *B. tenuissima* and in the mesophilic species *C. cornutum* is shown in **Figure 2.** The chains composition of DGCC and phospholipids was established according to Flaim et al. (2014). As an example, the MS/MS of DGCC 38:6 (*m/*z 953) showed fragments at *m/z* 544, attributable to the loss of 16:0, and at *m/*z 472, which is attributable to 22:6 fatty acid (**Figure 1B**). The fragmentation of DGCC under positive-ion ESI/MS/MS was less clear than that of galactolipids. Therefore, it was not possible to determine unambiguously their regiochemistry and, as an example, here DGCC 36:6 (16:0/22:6) only means that this molecular species is built on the two different acyl chains 16:0 and 22.6 but we don’t know which is where.

Among the betaine lipids (DGCC) and the phospholipids (PC), the molecular species 36:6 (14:0/22:6), 38:6 (16:0/22:6), and 44:12 (22:6/22:6) were the most abundant.

Less abundant lipid species, such as TGDG, PG, PE and SQDG, were not detected in all dinoflagellates. For example, PGs were not detected in *P. willei* and *P. cinctum*, PEs in *T. coronata* and in *C. cornutum*, and four SQDGs were only found in *P. aciculiferum*.

**FIGURE 3:**
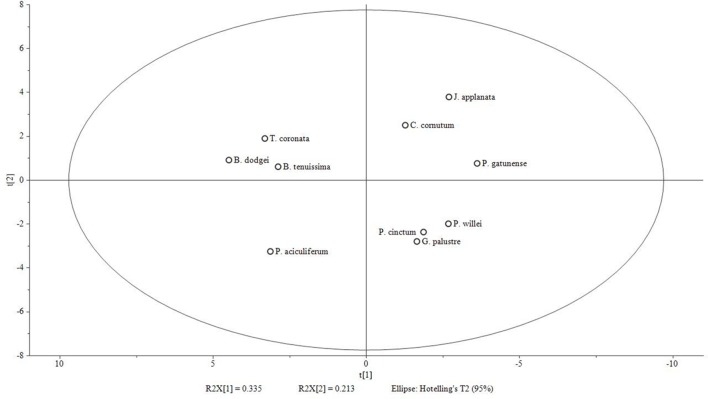
**Score plot of PCA model based on % area of fatty acids [log(x+1) transformed and Pareto scaled] generated with Simca-P 13.0 software.** 2 components; *R*^2^X (cumulative): 0.548; *Q*^2^ (cumulative): 0.104. Group 1 (left side of X-axis): *B. dodgei*, *B. tenuissima*, *P. aciculiferum* and *T. coronata*; group 2 (right side): *J. applanata*, *C. cornutum*, *P. gatunense*, *P. willei*, *P. cinctum*, *G. palustre.*

The first two principal components of the PCA based on % area of lipids explained 33.5 and 21.3% of the total variance, respectively. In the ordination based on the first principal component (**Figure 3**), the psychrophilic species *B. dodgei*, *B. tenuissima*, *P. aciculiferum* clustered with the mesophilic species *T. coronata* (cluster 1) versus all other species (cluster 2; *J. applanata*, *C. cornutum*, *P. gatunense*, *P. willei*, *P. cinctum*, *G. palustre*). The PLS-DA model with these two clusters as dependent variables was statistically significant (*Q*^2^ value = 0.836; *P* < 0.05). Based on the scatter plot of the corresponding OPLS-DA model, we identified metabolites that contributed to the separation between groups (**Table 1**). Group 1 was characterized by a higher percent of 36:9 (18:5/18:4) MGDG, 36:9 (18:5/18:4) DGDG and 36:6 (14:0/22:6) DGCC (**Table 2**). Group 2 was characterized by a higher percent of 38:9 (20:5/18:4) and 38:10 (20:5/18:5) MGDGs, 38:9 (20:5/18:4) and 38:10 (20:5/18:5) DGDGs, 32:1 TGDG, 34:1 SQDG, 32:1 PE and 34:2 PE (**Table 2**). Furthermore as investigated by a *t*-test comparing both groups, group 1 had a higher percent of total MGDGs than group 2, and group 2 had a longer AVLs of MGDG, DGDG and DGCC and a higher content of DGDG and TGDG than group 1 (**Table 3**).

**Table 2:** Marker lipids obtained by OPLS-DA analysis characteristic for clusters 1 and 2 indicated by principal component 1.

	Group 1				Group 2						
			
Markers	*B. tenuissima*	*B. dodgei*	*P. aciculiferum*	*T. coronata*	*C. cornutum*	*G. palustre*	*J. applanata*	*P. willei*	*P. cinctum*	*P. gatunense*	*P*-value
DGDG 36:9	79.1	82.3	81.1	77.4	1.4	17.9	n.d	1.7	13.0	10.8	5.02E–07
DGDG 38:9	n.d.	n.d.	n.d.	0.4	65.9	24.2	53.6	89.6	76.9	61.9	1.06E–03
DGDG 38:10	n.d.	n.d.	n.d.	n.d.	9.4	6.4	3.3	4.3	n.d.	2.7	2.11E–02
MGDG 36:9	29.7	45.1	42.2	43.3	2.9	9.5	n.d	6.4	n.d.	n.d.	6.68E–04
MGDG 38:9	2.2	n.d.	n.d	4.2	45.5	24.4	27.0	33.9	36.1	37.3	5.89E–05
MGDG 38:10	4.4	n.d	n.d	3.0	27.5	27.8	13.2	39.4	11.8	12.1	5.53E–03
TGDG 32:1	7.6	14.1	7.9	5.1	100.0	34.2	85.1	63.6	70.1	72.9	1.09E–03
DGCC 36:6	51.9	60.5	46.6	39.1	3.5	28.7	48.7	11.1	11.9	5.7	5.83E–03
SQDG 34:1	13.5	7.3	n.d	4.5	30.8	85.1	38.7	88.9	81.6	77.9	1.33E–03
PE 32:1	n.d.	n.d.	n.d.	n.d.	n.d.	41.6	34.3	64.1	n.d.	30.1	3.85E–02
PE 34:2	n.d.	n.d.	n.d.	n.d.	n.d.	27.8	37.9	35.9	100.0	41.1	2.93E–02


*Dinoflagellates are ordered with respect to their membership to clusters; given are % area of each lipid within a given lipid class and P-values of the Welch two sample t-test appropriate for unequal variances in clusters.*

**Table 3:** Lipid parameters for groups 1 and 2.

	Group 1				Group 2						
			
Parameter	*B. dodgei*	*B. tenuissima*	*P. aciculiferum*	*T. coronata*	*C. cornutum*	*G. palustre*	*J. applanata*	*P. willei*	*P. cinctum*	*P. gatunense*	*P*-value
DGDG (%)	53.6	54.2	52.9	55.1	80.0	65.6	62.9	72.6	62.1	68.2	3.43E–03
MGDG (%)	46.4	45.8	47.1	44.9	20.0	34.4	37.1	27.4	37.9	31.8	3.43E–03
[DGDG]/[MGDG]	1.2	1.2	1.1	1.2	4.0	1.6	1.7	2.6	1.6	2.1	
UI MGDG	9.1	9.6	9.4	9.2	9.1	6.2	9.3	9.6	9.7	9.6	N.S
UI DGDG	8.9	9.0	9.0	8.6	8.9	11.2	8.3	8.9	9.1	8.8	N.S
UI TGDG	1.8	1.8	6.5	2.4	1.0	3.2	1.0	1.3	1.2	1.0	N.S
UI DGCC	6.5	6.5	7.4	6.2	6.9	7.0	6.4	7.1	7.6	7.6	N.S
UI PC	9.0	9.0	9.4	9.5	12.0	9.2	8.6	9.2	8.5	9.7	N.S
UI SQDG	2.6	2.6	0.9	2.7	2.4	1.1	2.1	1.1	1.4	1.3	N.S
UI PG	3.7	3.8	0	3.4	3.0	2.0	3.0	0	0	3.0	N.S
UI PE	0	2.0	0	0	0	1.6	1.7	1.4	2.0	1.7	N.S
ACL MGDG	36.0	36.1	36.0	36.4	37.5	37.0	38.1	37.5	37.0	37.6	2.18E–04
ACL DGDG	36.0	36.0	36.0	35.9	37.3	36.6	37.6	37.9	37.5	37.5	2.13E–04
ACL TGDG	32.6	32.0	36.5	33.7	32.0	33.0	32.2	32.1	32.2	32.5	N.S
ACL DGCC	37.1	38.1	38.1	37.3	38.8	37.8	38.0	38.9	39.2	39.5	2.16E–02
ACL PC	40.0	40.3	39.9	41.0	44.0	40.6	40.1	40.8	40.0	41.9	N.S.
ACL SQDG	34.0	34.0	30.3	34.0	34.0	34.0	34.0	34.0	34.0	34.0	N.S
ACL PG	34.0	34.0	0	34.0	34	34.0	34.0	0	0	34.0	N.S.
ACL PE	0	34.0	0	0	0	32.6	32.8	32.7	34.0	32.8	N.S.


*Differences are investigated by the Welch two sample *t*-test appropriate for unequal variances in groups, and *P*-values of the *t*-test (N.S., not significant) are given. The percentage of each galactolipid class is given by the formula: % class_*x*_ = Σ (relative area of all lipids species belonging to class_*x*_ /Σ relative areas of all galactolipids); values were corrected for response factor obtained in ESI(+) MS. The UI of each lipid class, or the average number of unsaturation, is calculated using the formula UI_*class*_*_*y*_* = Σ (relative area of lipid*_*x*_*^∗^ double bond number of lipid*_*x*_*). The ACL of each lipid class, or the average length of acyl chains bound to specific lipid class, is given by the formula ACL_*class*_*_*y*_* = Σ (relative area of lipid*_*x*_*^∗^ acyl chain length of lipid*_*x*_*).*

**FIGURE 4:**
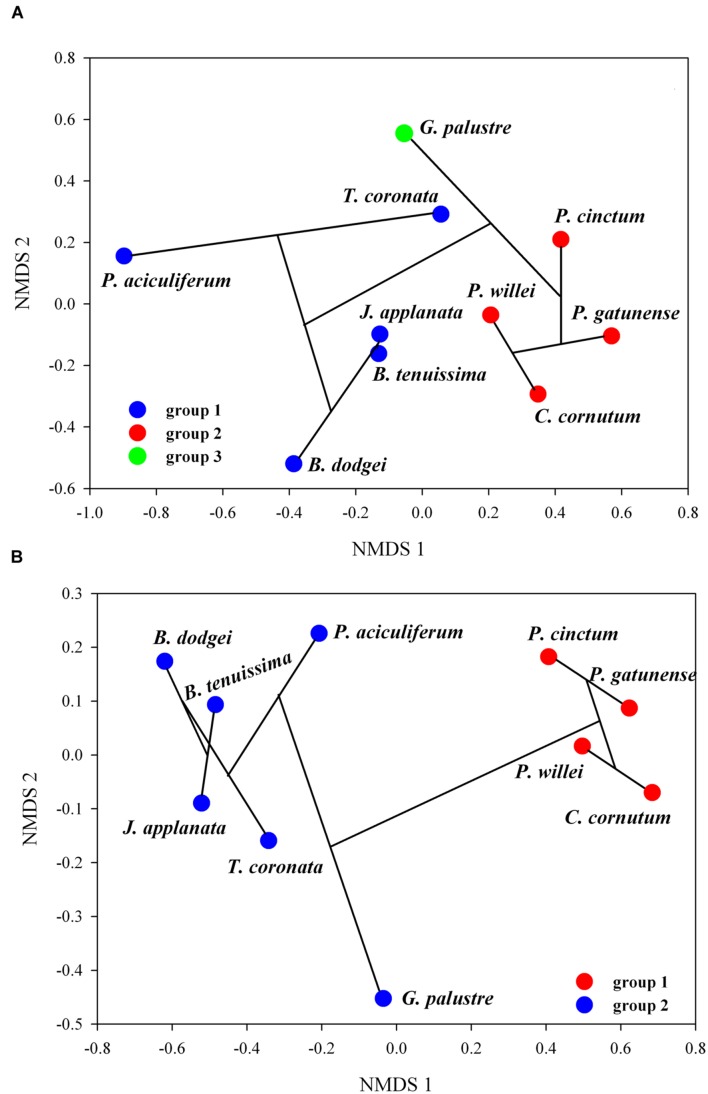
**Combination between hierarchical clustering and NMDS of betaine; shown is the NMDS ordination with clusters, indicated by bootstrap resampling, and the cluster dendrogram (the black lines connecting taxa): **(A)** % area of fatty acids, **(B)** UI**.

The correlation between the uncorrected genetic dissimilarity and the lipid profile dissimilarity based on % area and UI, respectively, was high for betaine (Mantel *r*_%area_ = 0.48, *P* = 0.004; Mantel *r*_UI_ = 0.49; *P* = 0.001). The correlation for PC was high (Mantel *r*_%area_ = 0.65, *P* = 0.03; Mantel *r*_UI_ = 0.67; *P* = 0.03) but was driven by *C. cornutum*; excluding this dinoflagellate, the correlation was not significant. For the other lipids the correlation was either non-significant (TGDG) or below 0.25 (MGDG, DGDG). For SQDG, the mantel correlation was non-significant based on % area but significant based on UI (Mantel *r*_UI_ = 0.34; *P* = 0.05); two influential distances between taxa determined this significant correlation and their exclusion led to an insignificant result (SQDG *r*_UI_ = 0.28; *P* = 0.09). To further investigate the relationship between genetic diversity and lipid profile by NMDS and cluster analysis, we only considered correlations >0.25 to focus on meaningful relationships. For betaine (% area), hierarchical clustering indicated three groups (*B. dodgei, B. tenuissima, P. aciculiferum, T. coronata, J. applanata* versus *P. cinctum, P. gatunense, C. cornutum, P. willei* versus *G. palustre*) (**Figure 4A**). This clustering was only partially evident in NMDS (*S* = 0.11) (**Figure 4B**). Based on the UI of betaine, in NMDS (*S* = 0.03) the former two large groups were supported by the clustering.

## Discussion

Temperature is one of the most important factors constraining life on earth (Litchman and Klausmeier, 2008). The active restructuring of the physical state of membranes in response to temperature changes, also known as the *homeoviscous* adaptation, is a very important aspect of temperature adaptation in poikilotherms (Hazel and Williams, 1990; Hazel, 1995). This is the first study that considers the intact molecular species of different membrane lipid classes (galactolipids, glycolipids, phospholipids and betaines) from ten freshwater dinoflagellates grown at specific temperatures akin to their natural occurrence. All dinoflagellates studied possessed the galactolipids MGDG, DGDG and TGDG, the glycolipid SQDG, the betaine DGCC and the phospholipid PC while the phospholipids PE and PG were not universally present. Lipid species that were not found in some dinoflagellates might have been under the detection limit of our experimental setup because of their low concentration. Thus, we focused our attention on meaningful concentrations and not on the absence of lipid species.

The freshwater dinoflagellates could be distinguished into two groups based on the regiochemical distribution of the major fatty acyl chains of MGDGs and DGDGs: the psychrophilic taxa *P. aciculiferum*, *B. dodgei*, *B. tenuissima* and the mesophile *T. coronata* belonged to the C_18_/C_18_ cluster while *C. cornutum*, *G. palustre*, *J. applanata, P. cinctum*, *P. willei* and *P. gatunense* belonged to the C_20_/C_18_ cluster *sensu* Gray et al. (2009b).

In our study, dinoflagellates of the C_18_/C_18_ cluster had a [DGDG]/[MGDG] molar ratio within a narrow range (1.1–1.2) while dinoflagellates of the C_20_/C_18_ cluster had a much higher ratio (1.6–4.0), further distinguishing these two clusters. The relative amount of galactolipids is important in temperature adaptation; MGDGs possess an inverted conical geometry and, therefore, in aqueous systems tend to form hexagonal-II phase rather than forming a bilayer (Gurr et al., 2002; Arouri and Mouritsen, 2013), and this can be an important property to sustain membrane fluidity with low temperature. In psychrophiles, MGDGs are more important for maintaining membrane fluidity than DGDGs (Flaim et al., 2012). Likewise in *Pyrocystis* spp., grown between 25 and 35°C, DGDGs respond more to temperature changes than MGDGs (Leblond et al., 2010a).

Furthermore, dinoflagellates of the C_18_/C_18_ cluster generally had a shorter acyl chain length with respect to C_20_/C_18_ cluster dinoflagellates. This is not surprising because many studies have confirmed the relationship between colder temperatures and shorter chain lengths (Gray et al., 2009b; Flaim et al., 2012, 2014) versus warmer temperatures and longer chain lengths (Adolf et al., 2007; Fuentes-Grünewald et al., 2012) in dinoflagellates.

While an increase in the UI with decreasing temperatures is commonly seen within a single species with changing temperature, both for warm- and cold-water dinoflagellates (e.g., Leblond et al., 2010a, 2015; Flaim et al., 2012, 2014), no differences in the UIs of all lipid classes were found for the C_18_/C_18_ and C_20_/C_18_ clusters. When the UI was applied to inter-specific comparisons, there was no difference between clusters; we suggest that this was driven by compensatory effects of single lipid species.

Photosynthetic organisms in general have different amounts of MGDG and DGDG species that change even when the degree of unsaturation of their associated fatty acid profile is not apparently modulated (Leblond et al., 2010a). We suggest that the mechanisms that control membrane fluidity seem to be very fine-tuned and species specific.

While Gray et al. (2009b) link the regiochemical clustering of species to habitat characteristics such as mixing and nutrients, here we give a physiological explanation. We suggest that the grouping of dinoflagellates in C_18_/C_18_ and C_20_/C_18_ clusters is actually an indication of the range of temperature tolerance of species and might separate stenotherms (i.e., organisms with a limited temperature tolerance) from eurytherms (i.e., organisms with a higher temperature tolerance). In the C_18_/C_18_ cluster, temperature tolerance seems to be limited, as indicated experimentally for the two psychrophile dinoflagellates *B. dodgei* (Flaim et al., 2012) and *P*. *aciculiferum* (Flaim et al., 2014). Warm-water dinoflagellates that belong to the C_18_/C_18_ cluster should therefore also have a limited temperature range (of ca. 10°C) that sustains optimum growth and metabolic functions. Indirect evidence of this can be found in several studies showing maximum values for different metabolic parameters in a narrow temperature range: *Scrippsiella trochoidea* (no difference in growth rates between 11 and 18°C with significant decreases at higher and lower temperatures; Binder and Anderson, 1987), no change in whole cell fluorescence in *Symbiodinium microadriaticum* between 24 and 28°C (Iglesias-Prieto et al., 1992), optimum growth of *Prorocentrum minimum* between 18 and 26°C (Grzebyk and Berland, 1996); furthermore, *Symbiodinium* spp. show different metabolite profiles at 18 and 26°C (Klueter et al., 2015) also indicating a narrow tolerance to temperature before significant changes occur.

Dinoflagellates belonging to the C_20_/C_18_ cluster instead seem to have a wider temperature tolerance, and thus thrive over a greater temperature range as indicated for *Alexandrium tamarense* (no change in survival rate between 0 and 25°C or 15 and 30°C depending on strain; Kobiyama et al., 2010) and *Pyrocystis lunula* (no difference in bioluminescence between 14 and 30°C; Craig et al., 2003). Furthermore, an *in situ* study on blooms of *Prorocentrum minimum* (C_18_/C_18_ cluster) and *Lingulodinium polyedrum* (C_20_/C_18_ cluster) indicated that the latter was not influenced by temperature while the former was negatively influenced by mixing depth (Shipe et al., 2007). We hypothesize that a shallow mixing layer indicates summer conditions and a narrower temperature range. The concept of temperature tolerance is eloquently shown by Butterwick et al. (2005), who carried out growth experiments with various algal species over a wide temperature range. Similarly to Butterwick et al. (2005), specific studies are needed to test our hypothesis on the extent of temperature tolerance in relation to the clustering of dinoflagellates. Dahmen et al. (2013) suggest that the C_20_/C_18_ cluster dinoflagellate *L. polyedrum* has specific elongases and desaturases to modulate the galactolipid chain length. Following the same corollary we speculate that differences in temperature tolerance between C_18_/C_18_ and C_20_/C_18_ clusters could be related to a different array of enzymes and/or their different activity and would thus define their eurythermic or stenothermic nature. Stenotherms originate from more thermally stable environments than eurytherms, and both stenotherms and eurytherms can better cope with temperature changes toward lower temperature than toward higher temperatures (Somero et al., 1996). Thus, understanding the mechanisms that underlie thermal adaptations is pivotal to predict how climate warming will affect species distribution (Somero, 2010).

While in recent years, the galactolipid composition in dinoflagellates has attracted attention (Leblond et al., 2006, 2010a; Gray et al., 2009a,b; Flaim et al., 2012) little information is available about betaine lipids (Leblond and Chapman, 2000; Flaim et al., 2014; Leblond et al., 2015).

This study corroborated that, in dinoflagellates, the FAC distribution of betaines remarkably differs from that of galactolipids: while the latter mainly possessed ω3 acyl chains such as 18:4, 18:5, and/or 20:5, predominant DGCCs possess short chain saturated fatty acids (14:0 – 16:0) together with long chain polyunsaturated one (22:6, ω3). Similarly to Flaim et al. (2014) and Leblond et al. (2015), 36:6 DGCC presented the highest percentage in dinoflagellates and was furthermore indicated as a marker for the C_18_/C_18_ cluster. Surprisingly, in the ordination specific for betaine lipids, the grouping in C_18_/C_18_ and C_20_/C_18_ cluster was not that evident; specifically, *J. applanata* and *G. palustre* clustered together with the C_18_/C_18_ cluster. This was also found for the C_18_/C_18_ dinoflagellate symbiont *Symbiodinium* (Leblond et al., 2015), where betaine lipids showed minimal changes with temperature. This unexpected result could be explained by the link between taxa lipid profile and genetic distance. Species within the groups *T. coronata* and *J. applanata* (Hansen et al., 2007; Daugbjerg et al., 2014), *B. tenuissima* and *B. dodgei* (Daugbjerg et al., 2014) and *P. gatunense*, *P. willei*, and *P. cinctum* (Logares et al., 2007) are close together in phylogenetic trees. We suggest that betaine lipids better reflected the phylogenetic relationship rather than the temperature tolerance in dinoflagellates. In fact, *J. applanata* and *G. palustre* showed the highest amount of 36:6 DGCC within the C_20_/C_18_ cluster and might well be grouped with the C_18_/C_18_ cluster.

In summary, our study indicated that the analysis of thylakoid glycolipids can shed light on the range of dinoflagellate temperature tolerance whereas the distribution of non-thylakoid lipids better reflects their phylogeny.

## Author Contributions

AA: lipid analysis and data analysis; UO and GF: dinoflagellate cultures, lipid extraction, data analysis; GH and AS: provided samples: GG: oversaw the research; all authors contributed to writing and approved the manuscript.

## Conflict of Interest Statement

The authors declare that the research was conducted in the absence of any commercial or financial relationships that could be construed as a potential conflict of interest.

## Supplementary Material

**TABLE S1 | Detailed information about lipid composition of each dinoflagellate species.** Columns report: (1) compound id (#); (2) Retention Time (RT; min) under chromatographic conditions reported in “Materials and Methods”; (3) Extracted Ion Chromatograms (EIC) for each lipid molecular species; (4) lipid class and parameters; (5) identity of lipid molecular species; (6) relative quantification (% area) in each dinoflagellate species.

## Abbreviations

ACL: average chain length
LC-ESI-MS: liquid chromatography-electrospray ionization- mass spectrometry
NMDS: non-metrical multidimensional scaling
OPLS-DA: orthogonal partial least square – discriminant analysis
PCA: principal component analysis
PLS-DA: partial least square – discriminant analysis
UI: unsaturation index
